# Individual cognitive therapy reduces frontal-thalamic resting-state functional connectivity in social anxiety disorder

**DOI:** 10.3389/fpsyt.2023.1233564

**Published:** 2023-12-21

**Authors:** Kohei Kurita, Takayuki Obata, Chihiro Sutoh, Daisuke Matsuzawa, Naoki Yoshinaga, Jeff Kershaw, Ritu Bhusal Chhatkuli, Junko Ota, Eiji Shimizu, Yoshiyuki Hirano

**Affiliations:** ^1^Research Center for Child Mental Development, Chiba University, Chiba, Japan; ^2^United Graduate School of Child Development, Osaka University, Suita, Japan; ^3^Institute for Quantum Medical Science, National Institutes for Quantum and Radiological Science and Technology, Chiba, Japan; ^4^Department of Cognitive Behavioral Physiology, Graduate School of Medicine, Chiba University, Chiba, Japan; ^5^School of Nursing, Faculty of Medicine, University of Miyazaki, Miyazaki, Japan

**Keywords:** individual cognitive therapy, neuromarker, resting-state functional connectivity, social anxiety disorder, thalamus

## Abstract

**Introduction:**

Previous neuroimaging studies in social anxiety disorders (SAD) have reported potential neural predictors of cognitive behavioral therapy (CBT)-related brain changes. However, several meta-analyses have demonstrated that cognitive therapy (CT) was superior to traditional exposure-based CBT for SAD.

**Objective:**

To explore resting-state functional connectivity (rsFC) to evaluate the response to individual CT for SAD patients.

**Methods:**

Twenty SAD patients who attended 16-week individual CT were scanned pre- and post-therapy along with twenty healthy controls (HCs). The severity of social anxiety was assessed with the Liebowitz Social Anxiety Scale (LSAS). Multi-voxel pattern analysis (MVPA) was performed on the pre-CT data to extract regions associated with a change in LSAS (∆LSAS). Group comparisons of the seed-based rsFC analysis were performed between the HCs and pre-CT patients and between the pre-and post-CT patients.

**Results:**

MVPA-based regression analysis revealed that rsFC between the left thalamus and the frontal pole/inferior frontal gyrus was significantly correlated with ∆LSAS (adjusted *R*^2^ = 0.65; *p* = 0.00002). Compared with HCs, the pre-CT patients had higher rsFCs between the thalamus and temporal pole and between the thalamus and superior/middle temporal gyrus/planum temporale (*p* < 0.05). The rsFC between the thalamus and the frontal pole decreased post-CT (*p* < 0.05).

**Conclusion:**

SAD patients had significant rsFC between the thalamus and temporal pole, superior/middle temporal gyrus, and planum temporale, which may be indicators of extreme anxiety in social situations. In addition, rsFC between the thalamus and the frontal pole may be a neuromarker for the effectiveness of individual CT.

## Introduction

Social anxiety disorder (SAD) is a mental disorder that features fear of evaluation by others in social situations (1). Reports suggest that the lifetime prevalence of SAD is not low, at 13% (2), and it is known to onset at a relatively young age, such as during adolescence, compared to other psychiatric disorders (3). According to the cognitive model of social anxiety proposed by Clark and Wells (4), patients with SAD have negative images of themselves and self-focused attention in social situations. Their social anxiety is maintained by internal focus and behaviors that temporarily make them feel “safe.” Pharmacotherapy (particularly selective serotonin reuptake inhibitors [SSRIs]) and psychotherapy (particularly individual cognitive therapy/cognitive behavioral therapy [CT/CBT]) are recommended for the treatment of SAD in clinical practice guidelines published from different countries (e.g., United Kingdom, Canada, Germany, Japan) (5–8). According to a meta-analysis (9), among various psychological and pharmacological options for intervention, individual CT/CBT is the most effective treatment. In the United Kingdom, individual CT/CBT is recommended as a first-line treatment option (3). In addition, when implementing CT/CBT, guidelines from the United Kingdom and Japan recommend individual treatment to patients over group-based treatment because the group format is less clinically and cost-effective than the individual format (5, 8). Although the guidelines clearly state treatment principles and options for SAD, it would be clinically beneficial to be able to predict the response to treatment before it is initiated.

It has been suggested that the pathophysiological mechanism behind the treatment of SAD depends on changing the activity in the amygdala to improve the symptoms (10, 11). Most neuroimaging studies of SAD focus on parts of the limbic system, such as the amygdala, insula, anterior cingulate gyrus, medial prefrontal cortex, and the typical fear circuit, which includes the striatum and thalamus (12). Regulating the response of the amygdala is thought to be important for the treatment of SAD (13), and the thalamus is thought to regulate the fear circuitry in the amygdala (14). Furthermore, the paraventricular nucleus of the thalamus (PVT) integrates signals related to threat and arousal and is the primary node transmitting to the cortical emotional network from the subcortical loop (15). Therefore, the PVT is an important node in the brain network for anxiety (16). As the gray matter volume of the right thalamus is much lower in SAD patients than in healthy controls (HCs), the possibility of abnormalities in the cortico-striato-thalamo-cortical (CSTC) circuit has also been mentioned (17). The pathophysiology of SAD (i.e., biased attention and negative images toward oneself) has also been reported to be associated with self-referential regions (e.g., hippocampus, parahippocampal cortex). SAD patients showed higher activation in self-referential regions during a self-referencing fMRI task compared to HCs (18).

Previous studies have been conducted to predict treatment response using demographic and clinical variables. Early onset, greater symptom severity, comorbidity with other anxiety disorders (including generalized anxiety disorder and simple phobia), and high expectations for the therapist’s role have been identified as potential predictive factors for lower efficacy of SAD treatment (19). The brain-based activity was better than baseline symptom severity in predicting which patients would improve (10, 20–23). Among the neuroimaging studies that have investigated CBT as a treatment for SAD, in most cases group CBT has been reported (10, 20, 24–27). There have been fewer reports of neuroimaging studies concerning individual CBT for SAD (11, 21, 28, 29). Previously, task-dependent MRI studies were unable to completely eliminate potential confounding factors associated with task performance. Therefore, in recent years, there has been a growing trend toward using resting-state functional magnetic resonance imaging (rsfMRI) in clinical neuroimaging research (30). In particular, rsfMRI could be useful for investigating the specific brain mechanisms involved in the clinical symptoms of SAD. Even though a large number of rsfMRI studies of SAD have been performed (30, 31), only one has reported on individual CBT (28). In a hypothesis-based study, Klumpp evaluated the severity of SAD and treatment response in regions of traditional interest (28). To avoid possible bias related to the hypothesis-based selection of regions of interest (ROIs), the present study was performed using multi-voxel pattern analysis (MVPA). Recent studies evaluating CBT response have reported that MVPA-based regression results were a strong predictor for treatment efficacy in comparison to evaluation using diffusion-weighted MRI fractional anisotropy (FA) and seed-based rsFC (10). Rather than using the resting-state functional connectivity (rsFC) of individual voxels, MVPA is a technique that identifies multivoxel rsFC patterns and uses them as independent variables. MVPA characterizes neural responses as patterns of connections between a voxel and every other voxel in the whole-brain (32). It has proved to be more informative about and more sensitive to the functional connectivity of the cortex than univariate analyses (33). MVPA also provides biomarkers to evaluate disease based on the observed functional connections (34).

All previous neuroimaging-based studies of SAD have focused on traditional CBT with exposure (e.g., Heimberg model) (35) and did not consider non-exposure-based CT (e.g., Clark and Wells model) (4). Exposure-based CBT and CT are the two most commonly used/recommended psychological interventions for SAD but, while there is some overlap between the underlying psychopathological models, many of the treatment components/techniques are distinct. CT for SAD employs cognitive/behavioral techniques to re-align the distorted cognitions with reality, including video feedback, attention training, and behavioral experiments manipulating attention and safety behaviors (4). Several treatment components/techniques used in traditional exposure-based CBT, such as repeated exposure to promote habituation, exposure hierarchies, and rating anxiety in feared situations, are not used in CT. Previous meta-analyses have reported that CT was superior to traditional exposure-based CBT in both short- and long-term outcomes for SAD (36), and individual CT based on the Clark and Wells model was the most cost-effective intervention among the various types of treatment for SAD (37). Thus, more neuroimaging studies focusing on the most effective psychological intervention for SAD (i.e., individual CT) are needed.

The main objective of the present study is to use rsFC to determine how resting brain function is altered by individual CT for SAD patients. We employed MVPA-based regression to assess individual CT effectiveness. After MVPA, regression analysis was performed using pre-CT rsfMRI data from SAD patients as an independent variable and the correlation with the post-CT Liebowitz Social Anxiety Scale (LSAS) was evaluated. In other words, can the rsFC in a specific region be used to predict the response of the individual CT? Furthermore, to understand the pathology of SAD, we compared the rsFC between groups (i.e., HCs vs. pre-CT and pre- vs. post-CT).

## Materials and methods

### Participants

This study was a secondary analysis based on data (not including HCs) from our previous randomized controlled trial evaluating the efficacy of individual CT for antidepressant-resistant SADs. Details of the study protocol of this randomized controlled trial have been published elsewhere (38, 39). As well as web-based and newspaper advertisements, patients with SAD were recruited by distributing posters and leaflets at medical institutions in Chiba Prefecture. SAD was diagnosed according to DSM-IV-TR (40), and those patients in the range 18–65 years old and with an LSAS score ≥ 50 were selected (38). The age of onset of SAD, comorbidity, and experience of antidepressants were confirmed for all patients. Only patients who had previously been treated with at least one course of SSRI, but were resistant to or intolerant of this medication (the mean number of previous courses of SSRI was 1.74 [range 1–3]), were included in this study (41). Exclusion criteria were substance abuse/dependence for at least 6 months prior to enrollment, psychosis, pervasive developmental disorder/intellectual disability, autism spectrum disorders, current high risk of suicide, antisocial personality disorder, any unstable medical condition, pregnancy, or lactation. In addition, we excluded patients if they reported “much” to “very much” improvement in the Clinical Global Impression Scale (CGI-S) (42) after 12 weeks of antidepressant medication before the start of this study. The pre-specified sample size for the original study was 42 (21 per study arm), which was set based on our pilot results (the estimated group difference in LSAS was 30 points [s.d. = 30], β = 0.8, α = 0.05). Twenty-one of the SAD patients were assigned individual CT in addition to their usual therapy. All patients were receiving their usual psychiatric care from their primary psychiatrist, but some of them (6/19, 31.6%) had discontinued taking antidepressant treatment at the beginning of this study. The remaining 21 SAD patients were placed in a treatment-as-usual (TAU) group. Although there were few dropouts from the TAU group for the original study, for the present report MRI data was necessary, so participants unable to undergo MR imaging were excluded, leading to nine dropouts from the TAU group. Consequently, we excluded the entire TAU group from the present study. The breakdown of reasons for dropout is as follows: three due to wire placement for dental orthodontics, two due to a tattoo, and one each for brain clip placement, claustrophobia, noise aversion, and metal placement after breast cancer surgery.

The study was performed at the National Institute of Radiological Sciences and the Department of Clinical Psychiatry of Chiba University Hospital. As well as the SAD patient data, 20 HCs, controlled for age, gender, education, and Wechsler Adult Intelligence Scale-Revised (WAIS-R), were also selected. With regards to the participation of healthy controls, we conducted interviews following a protocol similar to the Mini International Neuropsychiatric Interview (M.I.N.I) (43) and determined subject eligibility based on predetermined criteria. One patient was excluded from each of the individual CT and HC groups due to orthodontic wires, and 19 patients with SAD and 19 HCs underwent MRI scanning. One more HC was excluded due to failure to complete rsfMRI scanning.

Both the LSAS and the Beck Depression Inventory-II (BDI-II) (44) were used to evaluate the baseline severity of social anxiety and depression for all participants (Table 1). The Sheehan Disability Scale (SDS) was also measured. Anatomical T1-weighted imaging and rsfMRI were performed. An identical set of measures and MRI scans were performed after individual CT treatment for the SAD group. The CGI-S questionnaire was also completed by all patients post-treatment. The Japanese versions of all questionnaires used in this study have good reliability and validity.

**Table 1:** Demographic and clinical data.

	HC (*n* = 18)	SAD (*n* = 19)		Value of *p*	
		Pre	Post	HC versus Pre	Pre versus Post
Gender (female/male)	7/11	7/12	–	0.90^a^	–
Age (years)	30.91 ± 7.98	32.66 ± 8.65	–	0.52	–
Education (years)	15.39 ± 1.58	14.58 ± 1.61	–	0.13	–
WAIS-R	98.74 ± 14.81	96.02 ± 18.95	–	0.63	–
Age at onset of SAD (years)	–	18.00 ± 8.24	–	–	–
CGI-S	–	5.21 ± 0.98	2.53 ± 1.17	–	<0.001^d^
*Additional axis I diagnosis*					
Major depressive disorder	–	5		–	–
Others^*^	–	3		–	–
Concurrent antidepressant treatment at baseline	–	13		–	–
LSAS	31.72 ± 16.99	82.63 ± 21.66	38.21 ± 18.01	<0.001^b^	<0.001^b^
BDI-II	5.56 ± 4.57	23.53 ± 11.27	9.89 ± 9.05	<0.001^b^	<0.001^e^
SDS	2.00 ± 3.16	16.84 ± 6.01	9.11 ± 6.16	<0.001^c^	<0.001^e^

^a^Chi-square test, ^b^*t*-test with Bonferroni correction, ^c^Mann–Whitney U test with Bonferroni correction, ^d^Wilcoxon signed-rank test, ^e^Wilcoxon signed-rank test with Bonferroni correction. ^*^Including obsessive-compulsive disorder, bipolar II, and panic disorder. HC, healthy control; SAD, social anxiety disorder; WAIS-R, Wechsler Adult Intelligence Scale-Revised; CGI-S, Clinical Global Impression Scale-Severity; LSAS, Liebowitz Social Anxiety Scale; BDI-II, Beck Depression Inventory-II; SDS, Sheehan Disability Scale. Unless stated otherwise, mean ± SD is shown.

The studies involving human participants were reviewed and approved by the Institutional Review Board of Chiba University Hospital. The patients/participants provided their written informed consent to participate in this study.

### Treatment phase

The CT program was constructed based on the cognitive model of Clark & Wells (4). The core parts of CT include (a) developing an individualized version of the cognitive model of SAD; (b) conducting experiential exercises to demonstrate the adverse effects of self-focused attention and safety behaviors; (c) restructuring distorted self-imagery using video feedback; (d) practicing external focus and shifting attention; (e) conducting behavioral experiments to test the patient’s specific fearful concerns about social situations; (f) modifying worry and post-event processing; (g) surveying other peoples’ attitudes/opinions to issues that concern patients; (h) addressing remaining assumptions (schema work); (i) rescripting early memories linked to negative images in social situations; and (j) preventing relapse. Each session of individual CT lasted 50–90 min once a week for a total of 16 weeks. Seven therapists (a psychiatrist, four clinical psychologists, a nurse, and a psychiatric social worker) evaluated the treatment for SAD prior to the study. All of the therapists had completed the Chiba Improving Access to Psychological Therapies project: Chiba IAPT (45) as a training course, as well as attended training at an additional special CT workshop for SAD (14 h). All of the therapists attended a weekly supervision session, and the therapists’ competency was evaluated by the Chiba-IAPT supervisors using the Revised Cognitive Therapy Scale (CTS-R) (46). At the start of this study, the mean clinical experience and experience with individual CT/CBT of the therapists were 6.7 years (s.d.: 3.9) and 3.5 years (s.d.: 2.7), respectively. On average, each therapist treated 2.6 patients (range: 1–6) during the study. As the total mean CTS-R rating was 43.4 (range: 39–48), which was above the competence threshold (>36) expected in UK-CBT training programs, all the study therapists demonstrated an adequate level of competence in providing CT.

### Imaging data acquisition

MR imaging was acquired with a Siemens MAGNETOM Verio 3.0 T MRI scanner using a 12-channel head coil at the National Institute of Radiological Sciences. RsfMRI acquisition was performed with a gradient-recalled echo-planar imaging sequence (TR = 2000 ms, TE = 25 ms, flip angle = 75°, matrix = 64 × 64, thickness = 3.4 mm, voxel size = 3.44 × 3.44 × 3.74 mm^3^). The number of slices in each volume was 33, and 215 volumes were scanned in 7 min 10 s. All participants were instructed to keep still and to focus on the crosshairs projected onto a screen during scanning. T1-weighted imaging was acquired with a 3D magnetization-prepared rapid acquisition gradient-echo sequence (MPRAGE, TR = 2,300 ms, TE = 2.46 ms, flip angle = 9°, TI = 900 ms, matrix = 256 × 256, thickness = 1.0 mm, voxel size = 1.00 × 1.00 × 0.98 mm^3^).

### MRI data preprocessing

The default pipeline implemented in the CONN toolbox (32) of SPM 12 (Wellcome Trust Centre for Neuroimaging, London)^1^ running in MATLAB (The MathWorks, Inc.) was used to preprocess the functional imaging data. First, five scans were removed from the initial data to ensure that the magnetization had reached a steady-state value. Next, head motion correction was applied using the default functional realignment and unwarp routines. In functional outlier detection, outlier scans were identified from the observed global BOLD signal and the amount of subject motion in the scanner. Acquisitions with framewise displacement above 0.9 mm or global BOLD signal changes above five standard deviations were flagged as outliers (47). Detected outliers were excluded from this analysis. Functional slice-timing correction and functional outlier detection (percentile = 95%, global signal = 3, motion = 0.5) were also applied. Gray matter, white matter, and cerebrospinal fluid segmentation and normalization were performed with respect to the Montreal Neurological Institute (MNI) space. Finally, spatial smoothing was applied with a Gaussian filter of Full-Width at Half-Maximum (FWHM) of 8 mm. The influence of confounding factors and other noise from white matter and cerebrospinal fluid were removed with linear detrending. A band-pass filter (width 0.008–0.09) was applied to remove unwanted physiological motion. The cortex and subcortex were divided into 91 and 15 regions, respectively, according to the FSL Harvard-Oxford Atlas (48), and the cerebellum was divided into 26 regions using the Automated Anatomical Labeling atlas (49).

### Functional connectivity analysis

Using the CONN toolbox, MVPA was performed on the pre-CT data to extract regions associated with a change in LSAS (ΔLSAS) as CT effectiveness. First, pairwise connectivity patterns between each voxel and all other voxels were computed. Then, using the CONN default PCA, we reduced the dimensionality of the data by projecting the data from a higher dimensional space to a lower-dimensional subspace. The four strongest spatial principal components were selected based on an approximate 5:1 ratio between observations. PCA analyses are performed in the first-level voxel-to-voxel analyses. These analyses produce multiple outputs, including the individual subject-level maps and the variability and frequency of the time series (47). After significant components were identified, seeds were extracted as ROIs of the regions comprising the clusters based on the FSL Harvard-Oxford Atlas (48) for cortex and subcortical regions, and the Automated Anatomical Labeling atlas (49) for cerebellum regions. The regression analysis was performed as a *post hoc* test for second-level analyses of functional connectivity data to evaluate the significance of the rsFC as the independent variable and the pre-CT LSAS score as a covariate. Taking the mean signal time-course in each ROI as the seed, connectivity maps were made for all data sets by calculating the correlations between the seed and the time series of every other voxel in the brain. To consider the issue of the BDI-II score as a confounding factor, we conducted a sensitivity analysis after adding BDI-II as an independent variable to the regression model. Group comparisons of the rsFC between HCs and pre-CT patients (two-sample *t*-test) were then performed. Group comparisons of the rsFC between the SAD patients pre and post-CT (paired *t*-test) were also performed. The thresholds used to extract rsFC for further analysis were height threshold: *p* < 0.001 (uncorrected for multiple comparisons) and cluster threshold: *p* < 0.05 (familywise error (FWE) corrected for multiple comparisons).

### Statistical analyses

Statistical analyses were conducted using the IBM Statistical Package for Social Science 28.0.0 (SPSS).^2^ We tested the compliance of the data with normality using the Shapiro–Wilk test. For comparisons between the HCs and pre-CT groups, we adopted the two-sample *t*-test, and the Mann–Whitney U test was applied to compare variables that did not conform to a normal distribution. On the other hand, we adopted the paired t-test to test the normality of the pre-and post-CT group data, and we applied the Wilcoxon signed-rank test to compare variables that were not consistent with a normal distribution. Gender data were compared with a chi-squared test for proportion. The results of group comparisons for the LSAS, BDI-II, and SDS data were Bonferroni corrected at a level of *p* < 0.05.

## Results

### Clinical symptoms

No significant differences in patient age, gender, education, and the Wechsler Adult Intelligence Scale-Revised were found between the HCs and the SAD group pre-CT (Table 1). With regards to clinical symptoms, the SAD group pre-CT scored a mean LSAS of 82.63 (±: 21.66) and a mean BDI-II of 23.53 (±: 11.27), which were significantly higher than the scores of mean LSAS 31.72 (±: 16.99) and mean BDI-II 5.56 (±: 4.5) (*p* < 0.001) for the HC group.

The post-CT SAD patients showed significantly improved symptoms compared with their situation pre-CT, with a mean LSAS of 38.21 (±: 18.01) and a mean BDI-II of 9.89 (±: 9.05) (*p* < 0.001; Table 1). The SAD patients also showed a significant improvement in CGI-S (from a mean of 5.21 [s.d.: 0.98] to a mean of 2.53 [s.d.: 1.17]) and SDS (from a mean of 16.84 [±: 6.01] to a mean of 9.11 [±: 6.16]) (*p* < 0.001; Table 1).

### Regression analysis based on pre-CT rsFC

**Figure 1:**
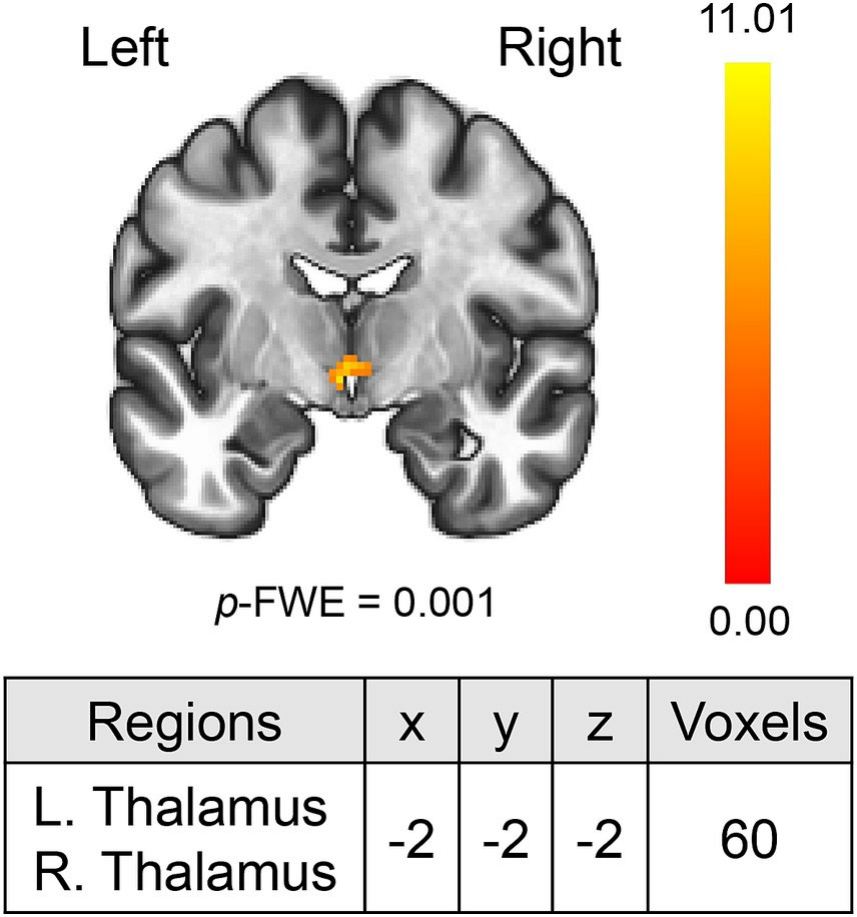
MVPA revealed the bilateral thalami which were correlated with ΔLSAS. MVPA, multi-voxel pattern analysis. The color bar indicates the *F* value.

**Figure 2:**
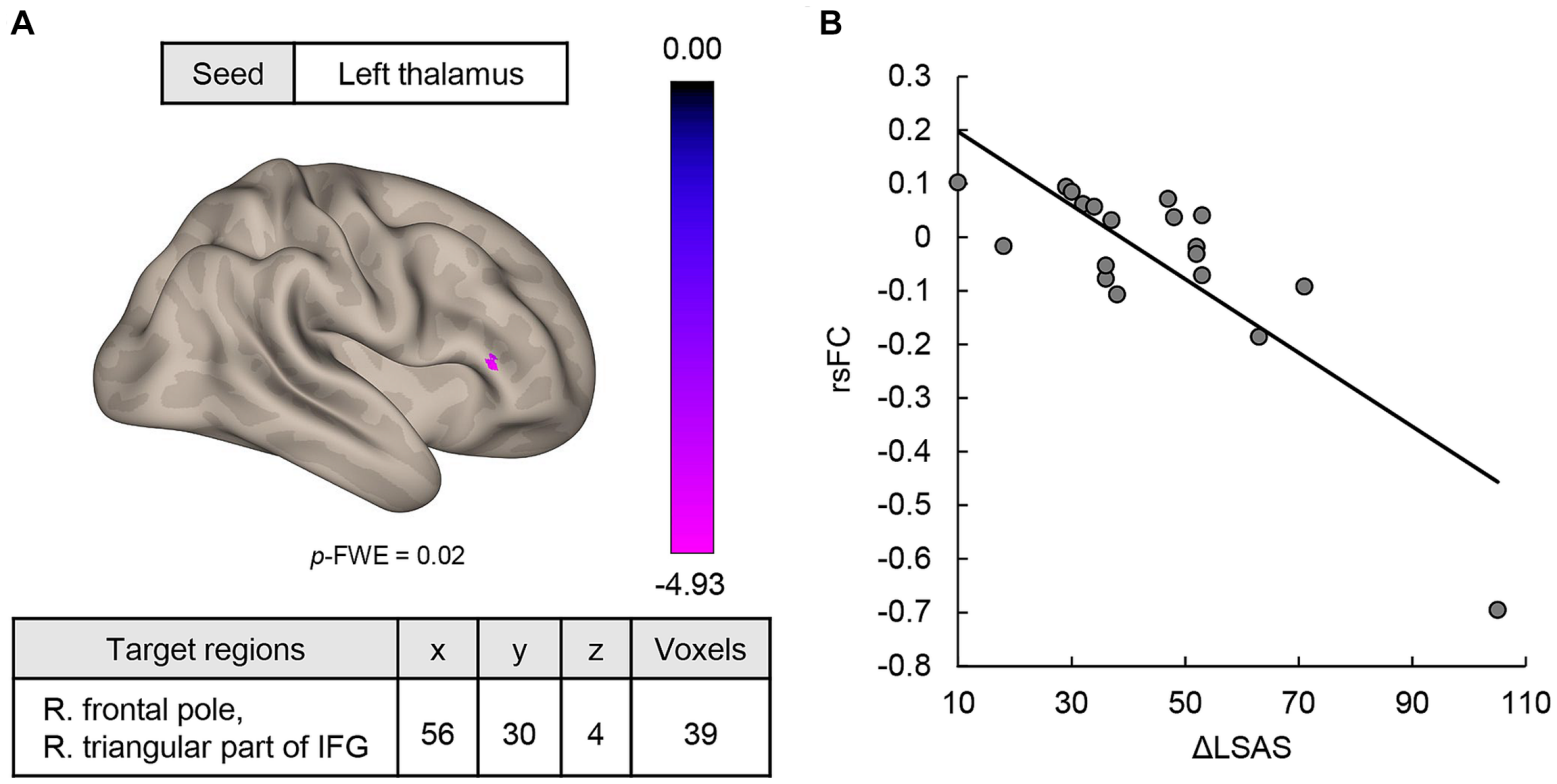
**(A)** MVPA-based regression analysis revealed regions where rsFC was correlated with ΔLSAS. Significant rsFC was found between the left thalamus and the right frontal pole/right triangular part of the IFG. **(B)** The rsFC and ΔLSAS were significantly negatively correlated. rsFC, resting-state functional connectivity; IFG, inferior frontal gyrus; LSAS, Liebowitz Social Anxiety Scale; cluster threshold: *p* < 0.05, familywise error (FWE) corrected for multiple comparisons. The color bar indicates the *F* value.

MVPA analysis revealed a significant cluster including two regions (bilateral thalami) associated with ΔLSAS (Figure 1). The cluster peak was located at [−2, −2, −2] and contained 60 voxels. Using the clusters as seeds, the results of the seed-based whole-brain rsFC analysis found significant rsFC between the left thalamus and right frontal pole/right triangular part of the inferior frontal gyrus (IFG; Figure 2A). The extracted rsFC was significantly correlated with the ΔLSAS (adjusted *R*^2^ = 0.65; *p* = 0.00002; Figure 2B). Prediction accuracy was also calculated (Supplementary Figure S1). Correlation between the rsFC and ΔLSAS remained significant after including BDI-II as a confounding factor (adjusted *R*^2^ = 0.63, *p* < 0.001). No significant rsFC was found using the right thalamus region as a seed.

### Comparisons of the rsFC for HCs versus pre-CT SAD patients

**Figure 3:**
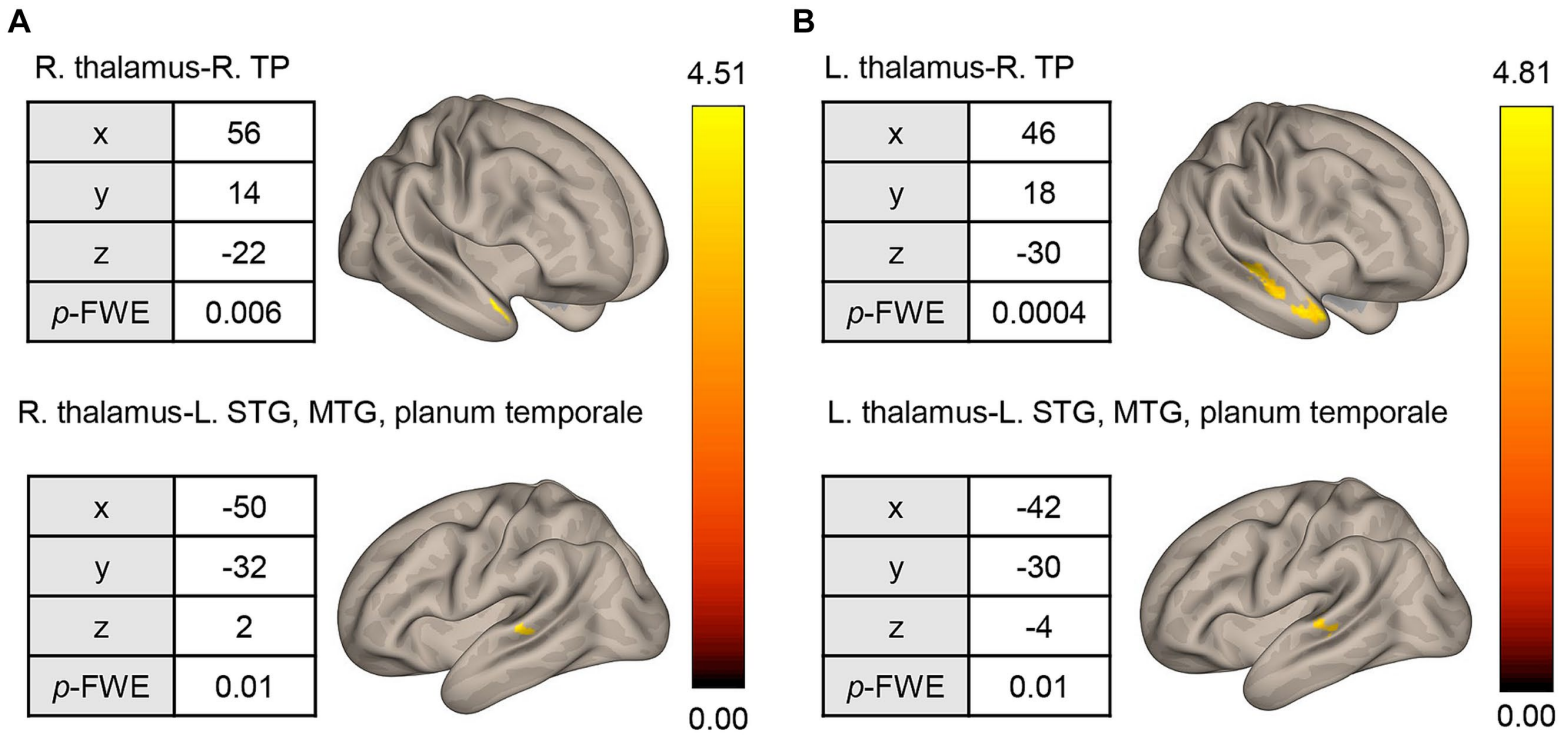
Seed-based whole-brain correlation analysis of differences in rsFC between the HC and pre-CT SAD groups. **(A)** Using the right thalamus as the seed, significant rsFC was found with respect to the right TP and STG/MTG/planum temporale. **(B)** Similar results were found when using the left thalamus as the seed. rsFC, resting-state functional connectivity; HC, healthy control; R, right; L, left; TP, temporal pole; STG, superior temporal gyrus; MTG, middle temporal gyrus; height threshold: *p* < 0.001, uncorrected for multiple comparisons; cluster threshold: *p* < 0.05, familywise error (FWE) corrected for multiple comparisons. The color bar indicates the *T* value.

The pre-CT SAD group showed higher rsFCs between the right thalamus seed and right temporal pole [56, 14, −22], and between the right thalamus seed and posterior part of the left middle temporal gyrus (MTG)/ posterior part of the left superior temporal gyrus (STG)/left planum temporale [−50, −32, 2] (Figure 3A). The former cluster contained 122 voxels, while the latter had 105 voxels. In addition, the rsFCs between the left thalamus seed and anterior/posterior part of the right MTG/STG [48, −18, −6], between the left thalamus seed and right temporal pole/anterior part of the right MTG [46, 18, −30], and between the left thalamus seed and posterior part of the left MTG/STG/left planum temporale [−42, −30, −4] were extracted (Figure 3B). The numbers of voxels in these clusters were 205, 179, and 106, respectively. Interestingly, one cluster (right temporal pole and left MTG, left STG, and left planum temporale) was common to both the left and right thalamus seeds (Figure 3). On the other hand, the pre-CT SAD group had a negative rsFC between the right thalamus seed and the left thalamus/left caudate nucleus [−10, −10, 14]. The cluster contained 143 voxels.

### Comparison of the rsFC for the pre and post-CT SAD groups

**Figure 4:**
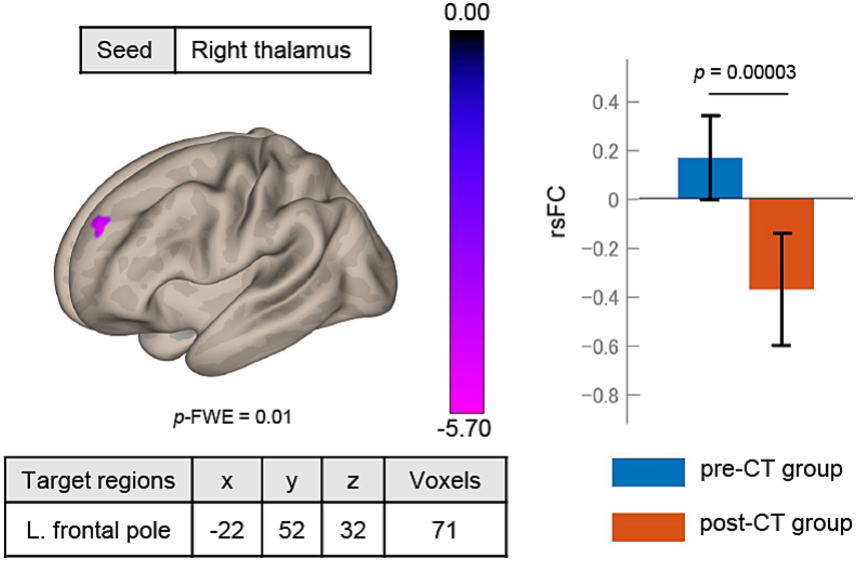
Changes in rsFC after treatment. The rsFC between the right thalamus seed and left frontal pole significantly decreased from pre-CT to post-CT. rsFC, resting-state functional connectivity; height threshold: *p* < 0.001, uncorrected for multiple comparisons; cluster threshold: *p* < 0.05, familywise error (FWE) corrected for multiple comparisons. The color bar indicates the *T* value.

The post-CT SAD group had lower rsFC between the right thalamus and left frontal pole [−22, 52, 32] than the pre-CT group (Figure 4). The cluster contained 71 voxels. No significant rsFC was found with the left thalamus seed region.

## Discussion

To our knowledge, this is the first study to analyze the relationship between rsFC and the response to individual CT for SAD patients. It was found that rsFC between the thalamus and frontal pole was significantly different before and after CT. Patients showed an improvement in depression and social anxiety symptoms after individual CT (Table 1). The rsFC between the left thalamus and right frontal pole/triangular part of the IFG may predict the ΔLSAS (Figure 2A). Characteristic of the rsFC of SAD among our study sample was that prior to CT, patients showed higher rsFC between the bilateral thalami and right temporal pole/MTG/STG/planum temporale than the HCs (Figure 3). The rsFC between the thalamus and frontal pole was significantly decreased after treatment with individual CT (Figure 4).

### Predicting treatment response by means of the rsFC between the thalamus and prefrontal cortex

The thalamus, identified by the MVPA as a region with a significantly non-zero ΔLSAS, is a component of the CSTC circuit. In SAD patients, the gray matter volume of the thalamus is much lower than in HCs, and the possibility of abnormalities in the CSTC circuit has been discussed (17). The thalamus is the primary node transmitting information from the subcortical loop to the cortical emotional network (15). In particular, the PVT is an important core region in the anxiety brain network, as it integrates signals related to threat and arousal (16). Furthermore, the PVT is thought to modulate fear processing in the amygdala (14). In a previous study of rsfMRI for individual exposure-based CBT, patients with higher connectivity between the amygdala and medial prefrontal cortex prior to treatment were more likely to benefit from CBT (28). In one fMRI study where a distress task was performed, an increase in thalamic activity was reported (50). The authors of that study suggested that the thalamus plays a role in regulating fear extinction and reward devaluation, and exposure may enhance these functions, leading to an improvement in the ability to positively regulate emotional signals.

With regard to the left and right thalamus, it is theorized that the right hemisphere is dominant for negative emotions (51). The pre-CT SAD group showed a significant lower rsFC between the left and right thalamus compared to the HCs. While it might be argued that thalamic connectivity can be interpreted based on the lateralization of brain function, a detailed interpretation is challenging based on the design of the present study.

The results of this study focused on the association between the left thalamus and the social anxiety scale. Another study similarly focusing on the left thalamus reported that connectivity with the left thalamus was associated with depressive and somatic symptoms (52). Previous studies have also reported that the thalamus is associated with depression and anxiety, and that it positively regulates emotions by fear extinction and modulating reward devaluation. However, further investigation is needed to determine left–right differences in function.

The frontal pole, which functionally connects with the thalamus, has been found to be involved in emotional and social cognitive processing (53). The triangular part of the IFG is also thought to be involved in emotional regulation (54, 55), and some dysfunction may lead to the downregulation of fear and anxiety responses (56). In this study, rsFC between the thalamus and frontal pole/triangular part of the IFG was correlated with the ΔLSAS, suggesting that SAD patients with less need of anxiety regulation for emotional and social cognitive processing respond better to individual CT. In addition, the rsFC between the thalamus and the prefrontal cortex decreased in the post-CT group. Together with the previous findings (50–53), our findings suggest that in SAD exposure increases thalamic activity, reducing the regulatory action of the prefrontal cortex, and leading to a decrease in rsFC between the thalamus and the prefrontal cortex. With respect to the cognitive model of SAD proposed by Clark and Wells, patients who perform safety behaviors to decrease anxiety are more likely to maintain social anxiety symptoms (4). In summary, SAD patients who received CT with exposure and without safety behaviors may enhance fear extinction and emotion regulation in the thalamus, leading to improved SAD symptoms.

### Characteristics of resting-state functional connectivity in SAD patients compared to HCs

From the comparison of the pre-CT patients and HCs, the temporal pole/posterior MTG/STG/planum temporale area had significant rsFC with both the left and right thalamus seeds. These regions are all involved in social cognitive function, which is also called the ‘Social Brain.’ Hyperactivity in social cognitive regions has previously been observed in SAD patients compared to HCs (57). The temporal pole receives various sensory inputs from the limbic system and serves as a hub for socioemotional cognitive functions (58). It is also part of the default mode network (DMN) and is also well-known as a self-referential region. With regard to psychiatric disorders, it is thought that the DMN, central executive network and salience network may be involved in the causation of symptoms (59). In SAD patients, the gray matter volume of the temporal pole is relatively small (60), and higher activation was reported for that region in a self-referencing task compared to HCs (18).

In the present study, the temporal pole formed a higher rsFC with the thalamus, which is part of the anxiety regulation network, suggesting that SAD patients experience more anxiety in a self-referencing task than HCs and are trying to regulate it. The other clusters, posterior MTG, STG, and planum temporale, are in regions that are anatomically peripheral to the superior temporal sulcus (STS). The STS selectively integrates visual and auditory stimuli, such as facial expressions and eye contact, to understand and interpret the behavior of others (61). In particular, the posterior STS (pSTS) is strongly involved in social interactions (62) and is an important brain region that activates as a cue for social cognition (63). In this study, rsFC between the thalamus and pSTS was higher for pre-CT patients than for HCs. Therefore, SAD patients may be more strongly anxiety modulated than HCs in social cognitive situations in response to facial expressions and eye contact. Furthermore, the temporal pole and pSTS clusters extracted in this study form a network with the medial prefrontal cortex and other regions. The temporal pole and pSTS are also involved in trying to understand the minds of others (64). In summary, the characteristics of SAD may be due to self-reference anxiety in social cognitive situations, which can reduced by individual CT.

### Limitations

In this study, rsFC between the thalamus and the prefrontal cortex was strongly correlated with ΔLSAS. Our results suggest that this rsFC metric could serve as a potential predictor of CT response. However, further research with a larger cohort is necessary to verify this hypothesis. The sample size of this study was relatively small as only 21 SAD patients participated.

Our study did not implement exposure-based CBT conditions or other psychological placebo conditions as a control. Therefore, we could not determine whether it was individual CT by itself or other factors (including general CBT techniques and natural temporal trends) contributed to the decreased rsFC between the thalamus and the frontal pole observed in this study.

SAD patients with psychiatric comorbidities were not excluded. In fact, several patients showed symptoms of depression as well as social anxiety that may have affected the BDI-II assessment. It was also difficult to control the usage of antidepressants while the CT was ongoing, and 31.6% of the participants discontinued taking antidepressant treatment before the study. The heterogeneity of participant backgrounds and concurrent treatment might have affected the treatment outcomes observed in this study. However, to create conditions under which we could best observe the influence of CT, we chose patients with low treatment responses to antidepressants and restricted medication change.

Our analysis took advantage of MVPA-based regression analysis. However, the utilization of regression analysis alongside group analyses resulted in diminished statistical power. Another limitation is the issue of parcellation of the thalamus. The bilateral thalami was extracted as a cluster by MVPA in this study. The analysis was performed using the cluster as a seed, but the actual nucleus of the thalamus is anatomically subdivided. We did not parcellate the neuroimaging data.

## Conclusion

To our knowledge, this is the first study to apply rsFC-based analysis to assess the effectiveness of individual CT for SAD patients. The severity of symptoms improved after individual CT. Comparing the HC and SAD groups, the latter had higher rsFC between the thalamus and MTG/STG/planum temporale, suggesting strong anxiety regulation through compensatory mechanisms in social cognitive situations. The results also showed that rsFC between the thalamus and frontal pole decreased post-CT. Furthermore, individual CT including exposure is expected to heighten activity in the thalamus and lead to a reduction in regulation by the frontal cortex, resulting in decreased connectivity between the thalamus and frontal cortex in SAD. The rsFC between the thalamus and frontal pole may be a neuromarker for the effectiveness of treatment.

## Data availability statement

The datasets presented in this article are not readily available because sharing the dataset is not permitted by the ethics committee. Requests to access the datasets should be directed to YH, hirano@chiba-u.jp.

## Ethics statement

The studies involving humans were approved by the Institutional Review Board of Chiba University Hospital. The studies were conducted in accordance with the local legislation and institutional requirements. The participants provided their written informed consent to participate in this study.

## Author contributions

YH, NY, ES, and TO designed the study. NY and ES recruited patients and supervised the psychotherapy. YH conducted the intelligence test and screening for MRI scanning. YH, CS, DM, and TO performed the experiment and collected data. KK and YH analyzed the data and drafted the manuscript. KK, YH, NY, ES, RC, JO, TO, and JK interpreted the data and reviewed the manuscript. All authors contributed to the article and approved the submitted version.

## Footnotes

^1^ www.fil.ion.ucl.ac.uk/spm/

^2^ www.spss.com
