# Supplementary material for: Individual cognitive therapy reduces frontal-thalamic resting-state functional connectivity in social anxiety disorder
Source: Front Psychiatry. 2023 Dec 21;14:1233564. doi: 10.3389/fpsyt.2023.1233564 (PMC10764569; doi:10.3389/fpsyt.2023.1233564)
Supplement: Supplementary file 1 [file Data_Sheet_1.docx]

Supplementary Material

# Statistical analysis

In order to compare the prediction accuracy, we performed multiple linear regression with Liebowitz Social Anxiety Scale (LSAS) pre-cognitive therapy (CT) (LSAS-pre) and pre-CT resting-state functional connectivity (rsFC), as independent variables. The FC was extracted with seed-based regression analysis. The prediction performance was validated using leave-one-out cross-validation (LOOCV). This process involves training the model using all subjects but one and this was repeated until all the subjects has been left out at least once. This ensures the prevention of overfitting of the data and generalizes the prediction. Pearson’s correlation coefficient between LSAS-pre and ΔLSAS was computed for the social anxiety disorder (SAD) patients.

# Supplementary results

According to Pearson’s correlation, the LSAS-pre was positively and significantly (*r* = 0.63; *p* = 0.003) correlated with ΔLSAS (Supplemental Figure 1a). Regression analysis using only LSAS-pre shows low variance (adjusted *R*^2^ = 0.09) (Supplemental Figure 1b). The results from LOOCV on FC between the thalamus and frontal pole/triangular part of inferior frontal gyrus (IFG) and LSAS-pre as independent variables (adjusted *R*^2^ = 0.56; *p*<.05) (Supplemental Figure 1c). Regression analysis using only the FC, without LSAS-pre is almost equivalent to the model with LSAS-pre in it (adjusted *R*^2^ = 0.57; *p*<.05). The addition of other parameters such as gender and age to the regression analysis did not increase the amount of variance (adjusted *R*^2^ = 0.31; *p*<.05).

**
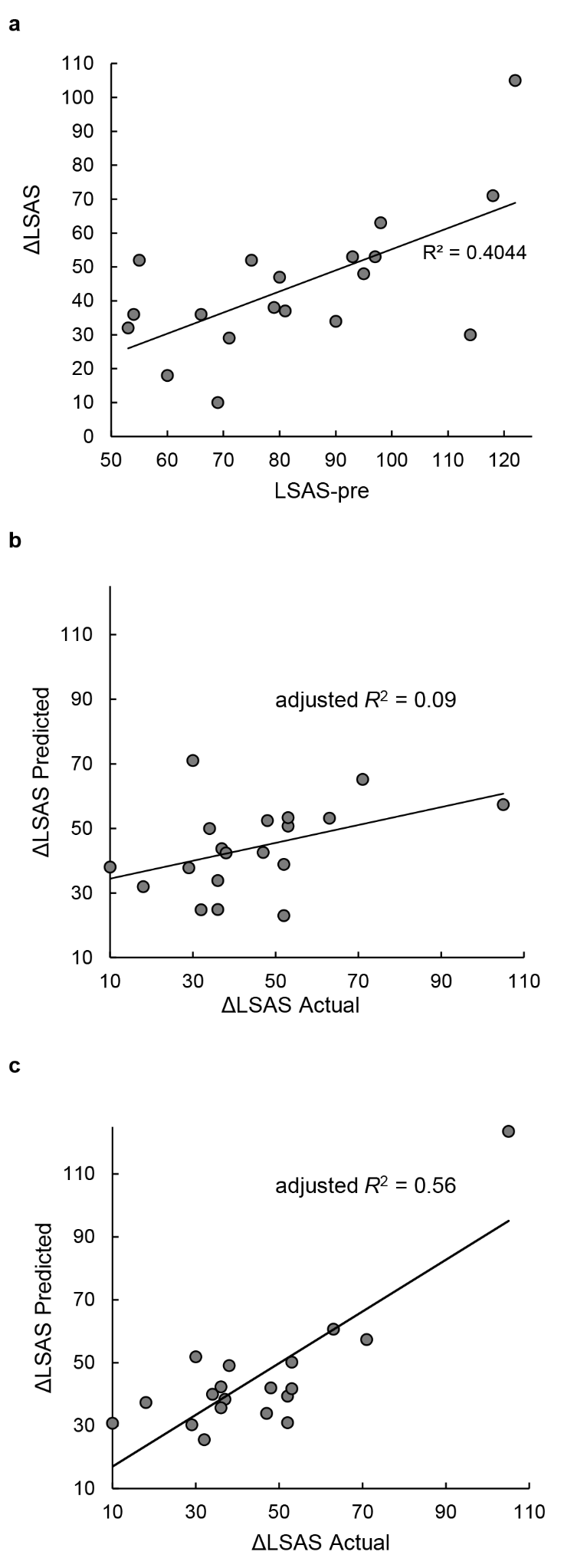
**

**Supplementary Figure 1.** Correlation and prediction of Liebowitz Social Anxiety Scale (LSAS) scores. (a) Relation of LSAS in pre-CT to CT effectiveness (ΔLSAS). (b) Relation between predicted ΔLSAS from multiple linear regression analysis using LSAS in pre-CT and subject information. (c) Relation between predicted ΔLSAS using leave-one-out cross-validation (LOOCV) and actual LSAS-change. LOOCV using the FC between the thalamus and frontal pole/ triangular part of inferior frontal gyrus and LSAS pre-CT SAD groups (LSAS-pre).
